# Orchid Mycorrhizal Association of Cultivated *Dendrobium* Hybrid and Their Role in Seed Germination and Seedling Growth

**DOI:** 10.3390/microorganisms12061176

**Published:** 2024-06-10

**Authors:** R. M. S. Ruwan Chamara, Kazumitsu Miyoshi, Tomohisa Yukawa, Nobuyuki Asai, Yuki Ogura-Tsujita

**Affiliations:** 1 The United Graduate School of Agricultural Sciences, Kagoshima University, Korimoto, Kagoshima 890-8580, Japan; ruwanchamara034@gmail.com; 2 Faculty of Agriculture, Saga University, 1 Honjyo-Machi, Saga 840-8502, Japan; 3 Graduate School of Horticulture, Chiba University, Matsudo 271-8510, Chiba, Japan; miyoshi@chiba-u.jp; 4 Tsukuba Botanical Garden, National Museum of Nature and Science, Amakubo, Tsukuba 305-0005, Japan; yukawa@kahaku.go.jp; 5 Asai Taikeien, Higashiura 470-2103, Aichi, Japan

**Keywords:** horticultural hybrids, orchid cultivars, symbiotic culture, Tulasnellaceae, wood-decay fungi

## Abstract

Orchids are crucial for the horticulture industry. Mycorrhizal fungi benefit crops by improving nutrition, plant growth, and disease resistance. However, the mycorrhizal association of horticultural hybrid orchids is poorly understood. To address this, we investigated mycorrhizal colonization in the entire root system and assessed the mycorrhizal community using a *Dendrobium* cultivar, *D*. Stardust ‘Firebird’, obtained from three nurseries. Additionally, we isolated and tested mycorrhizal fungi in symbiotic culture to assess their role in the seed germination and growth of *Dendrobium* species. All plants were colonized by mycorrhizal fungi, with a higher colonization rate in mature than in juvenile plants. Molecular identification of mycorrhizal fungi by Sanger and high-throughput sequencing revealed that the cultivar was associated with a phylogenetically diverse group of fungi, including mycorrhizal fungi from Tulasnellaceae, and several wood-decaying fungi. The Tulasnellaceae isolates significantly enhanced the seed germination of three *Dendrobium* species and increased the survival rate and growth of asymbiotic seedlings of *D. moniliforme*. This study is the first comprehensive examination of mycorrhizal associations in horticultural orchid hybrids, providing valuable insights for commercial production.

## 1. Introduction

Orchids are important in the global floriculture industry due to their aesthetic appeal and longevity, and numerous hybrid cultivars have been bred from economically important genera, such as *Dendrobium*, *Cattleya*, and *Phalaenopsis* [1,2]. Orchidaceae plants have established a unique symbiotic relationship with specific fungi for nutrient supply [3]. Their tiny seeds lack nutritional reserves and require carbon from mycorrhizal fungi for germination and growth under field conditions [4]. These fungi persist in adult orchids’ roots and/or rhizomes, providing nutrients and water to the host [3]. Wild orchid species, whether terrestrial or epiphytic, are exclusively associated with mycorrhizal fungi. Although the mycorrhizal fungi may play a role in commercially produced orchid hybrids, little research has been conducted on their mycorrhizal associations.

Horticultural orchids, many of which have complex hybrid origins, are typically propagated clonally by in vitro micropropagation methods using nutrient-rich media without fungi [5]. The resulting asymbiotic plantlets are acclimatized and grown in pots within greenhouses. This implies that the mycorrhizal association of horticultural cultivars may begin during vegetative growth, whereas wild orchids initiate this association at the seed-germination stage. The application of mycorrhizal fungi to asymbiotic orchid seedlings increases their survival rate [6], promotes plant growth and flower size [7], and enhances plant disease resistance [8]. Therefore, understanding mycorrhizal associations in industrial orchids could benefit orchid cultivation; however, our knowledge in this area is limited.

Partial mycorrhizal colonization has been observed in multiple orchid cultivars in nurseries in New Zealand [9] and Singapore [10]. However, previous studies examined only a few aspects of root length and number; the overall colonization rates of whole root systems are unknown. Furthermore, sampling has been restricted to a single cultivar from a single nursery. Obtaining samples from multiple nurseries and various growth conditions is essential to determine whether the mycorrhization of orchid cultivars is unique to a particular environment.

Wild orchids typically host highly diverse orchid mycorrhizal fungi (OMF) [11,12], mainly belonging to the Basidiomycota families Tulasnellaceae, Ceratobasidiaceae, and Serendipitaceae [4]. However, the composition of OMF communities in horticultural hybrids is unclear, although Tulasnellaceae fungi are often found in hybrid cultivars such as *Vanda* [13], *Cymbidium*, and *Dendrobium* [14]. Sampling from multiple nurseries would enable an accurate assessment of the mycorrhizal communities of a cultivar and their spatial distribution. Because most hybrid orchids are clonally propagated, they are genetically unique. Therefore, differences in OMF communities among nurseries could result directly from cultivation conditions or environmental variations. A comparison of OMF communities of a single cultivar among multiple nurseries would be a good model to evaluate the effects of environmental conditions on the OMF community.

In orchid cultivars, the nurseries responsible for plantlet establishment typically differ from those where they mature. For instance, tropical Asian nurseries export micropropagated seedlings after potting. Most Japanese orchid nurseries import these potted seedlings and grow them to flower in greenhouses. The variation in fungal communities with changes in the nursery environment is an intriguing aspect of the mycorrhizal associations of horticultural orchids. In addition, the functions of OMF infecting horticultural cultivars are unknown. OMF can have diverse functions; some fungal species promote seed germination and subsequent growth, while others do not affect seed germination but support leafy seedling growth [15,16]. Conducting symbiotic culture experiments using orchid seeds and asymbiotic seedlings would provide insights into these functions.

To investigate the mycorrhizal associations of commercially produced orchids, we addressed three questions: (1) What is the colonization rate of the entire root system? (2) Which fungal species colonize the horticultural cultivar? (3) Do OMF from the cultivar facilitate seed germination and plant growth? We investigated a horticultural orchid hybrid, *Dendrobium* Stardust ‘Firebird’. We examined mature plants from two Japanese nurseries and potted juvenile plants obtained from a nursery in Thailand. For the first question, we assessed fungal infection throughout the root systems of all individual plants. For the second question, we molecularly identified orchid mycorrhizal fungi by Sanger and high-throughput sequencing (HTS). Although HTS can detect a more diverse fungal community than Sanger sequencing, several Tulasnellaceae lineages may not be detected because of primer bias, even when using Tulasnellaceae-specific primers [17]. We used two Tulasnellaceae-specific primers for HTS to address this bias and identified the same samples using the Sanger method. For the third question, we isolated and identified fungal strains from the cultivar roots and subjected them to in vitro seed germination tests. We also inoculated these strains into asymbiotic leafy seedlings to examine the effect on leafy seedling growth. The findings provide comprehensive information on mycorrhizal associations in horticultural orchid cultivars.

## 2. Materials and Methods

### 2.1. Study Species and Sampling

**Figure 1:**
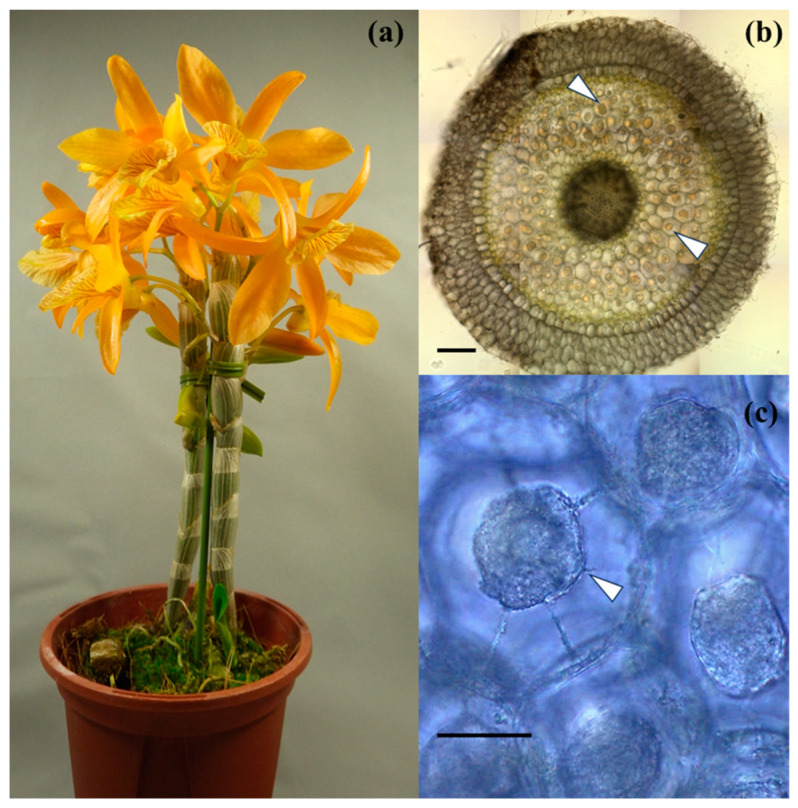
(**a**) Blooming mature *Dendrobium* Stardust ‘Firebird’. (**b**) Transverse sections of *D*. Stardust ‘Firebird’ root; the cortical area is fully colonized by intracellular hyphal coils (pelotons, indicated by arrows). Bar 100 µm. (**c**) Fungal peloton (arrow) in cortical cells. Bar 30 µm.

*Dendrobium* Stardust ‘Firebird’ is a popular orchid hybrid known for its vibrant orange flowers that emit a pleasant, sweet fragrance during spring bloom (Figure 1a). This hybrid originated from the cross between *D. unicum* (seed parent) and *D*. Ukon (pollen parent). This hybrid was created from five primary ancestors, each contributing varying proportions of the genetic composition—50% from *D. unicum*, 25% from *D. moniliforme*, 12.5% from *D. nobile*, and 6.25% each from *D. signatum* and *D. heterocarpum*. We obtained eight mature plants, each aged 3 years after potting, from nurseries in Aichi and Kumamoto Prefectures in Japan (separated by approximately 650 km). Additionally, we imported five juvenile plants, each aged 2 years after potting, from a nursery in Chiang Mai Province, Thailand (Table 1).

### 2.2. Assessment of Mycorrhization

To evaluate mycorrhization, we examined hand-cut sections covering the entire root system of each plant. First, we removed medium particles adhering to the roots by gently rinsing them with running tap water. Subsequently, we horizontally divided each root into 2 cm segments, starting from the base. Two or three hand-cut sections per truncated root segment were examined under a dissecting microscope (Nikon 50i, Tokyo, Japan) to assess mycorrhizal colonization. A root segment was considered mycorrhizal if at least one peloton was observed in any slice, irrespective of peloton quantity. Conversely, segments were classified as nonmycorrhizal if no mycorrhizal formation was detected in any root slice. Colonization frequency was calculated as (number of colonized root sections/total number of root sections examined) × 100. Colonization intensity was scored on each section using a six-step scale representing 0, 12.5%, 25%, 50%, 75%, and 100% of the cortical area (Figure 1b,c) [18]. Colonization frequency was assessed by χ2 test, and colonization intensity across various locations was compared by analysis of variance (ANOVA) followed by Tukey’s test in the R statistical package (v. 4.2.1) [19]. Root slices with living pelotons were used for fungal isolation.

### 2.3. Isolation of Mycorrhizal Fungi

Root fragments containing living pelotons were washed in sterile distilled water (SDW) to remove debris from the root surface. Individual pelotons were released from cortical cells into fresh SDW using a scalpel and tweezers under a stereomicroscope. Pelotons were collected and rinsed three times with SDW using a micropipette. Next, pelotons with 20–30 µL of SDW were added to a 9 cm sterile Petri dish containing 1.5% water agar medium (10 mL) with streptomycin and tetracycline (50 ppm each). The plates were incubated at 25 ± 1 °C for 1 week, and fungal colonies formed from individual pelotons were transferred to fresh potato dextrose agar (PDA) plates for subculturing. Pure cultures of OMF were obtained and identified using molecular methods, as described in the following section. Isolates were stored at −80 °C until use for symbiosis experiments and deposited with the National Institute of Technology and Evaluation (NITE) Biological Resource Center (NBRC) in Japan under accession numbers NBRC116648 and NBRC116574.

### 2.4. DNA Extraction, PCR, and Sanger Sequencing

Colonized root fragments were washed with TE buffer under a stereomicroscope and crushed with forceps to disperse hyphal coils into a fresh buffer. Pelotons (100–300 per root fragment) were collected using a micropipette, rinsed twice in fresh buffer, and homogenized in 20 µL of buffer using a BioMasher II homogenizer (Nippi Inc., Tokyo, Japan). For fungal isolates, hyphae growing on culture medium were collected using a sterilized toothpick and suspended in 50 µL of TE buffer. DNA extraction was conducted as described by Izumitsu et al. [20]. The supernatant was used as a template for polymerase chain reaction (PCR).

PCR was performed to amplify the ITS sequence of nuclear ribosomal DNA using the universal primers ITS1F/ITS4 [21,22] and ITS1F/ITS4B [22]. Additionally, because of the difficulty in amplifying the ITS region of the common OMF Tulasnellaceae using universal fungal primers [23], we used the Tulasnellaceae-specific primers ITS5/ITS4-Tul2 [21,24] and 5.8S-Tulngs/ITS4-Tul2 [24,25]. PCR amplification was conducted using Mighty Amp DNA polymerase v. 3 (TaKaRa, Shiga, Japan), following the protocol described by Chamara et al. [15].

PCR amplicons were purified using a FastGene Gel/PCR Extraction Kit (Nippon Genetics, Tokyo, Japan) and sequenced using a BigDye Terminator v. 3.1 Cycle Sequencing Kit (Thermo Fisher Scientific Baltics, Vilnius, Lithuania) and a 3130 Genetic Analyzer (Applied Biosystems, Tokyo, Japan), following the manufacturer’s instructions. To determine the taxonomic identification of putative OMF, we searched the GenBank database of the National Center for Biotechnology Information (NCBI) for the ITS sequences using the Basic Local Alignment Search Tool (BLAST). Next, fungi were assigned to operational taxonomic units (OTUs) based on 97% sequence similarity, and OTU numbers were allocated based on the OMF database in our laboratory. Subsequently, the full-length ITS sequence of each OTU was edited using ATGC v. 7 sequence assembly software (Genetyx, Tokyo, Japan) and deposited in the DNA Data Bank of Japan (DDBJ) under the accession numbers LC814993–LC815000.

### 2.5. Phylogenetic Analysis

A phylogenetic analysis of putative mycorrhizal fungi in the Tulasnellaceae family was conducted using the four OTUs identified in this study. Representative fungal ITS sequences from GenBank that showed a sequence similarity of >97% to the OTUs were included in the analysis. Nuclear ribosomal DNA sequences were aligned using the MAFFT online server [26,27]. Positions with <90% site coverage were eliminated, i.e., fewer than 10% alignment gaps, missing data, and ambiguous bases were allowed at any position. The TN93+G was estimated to be the best-fit model in MEGA11. The phylogenetic tree was constructed using the maximum-likelihood method in MEGA 11 [28]. Bootstrap analysis was performed with 1000 replicates to estimate the relative robustness of branches in the phylogenetic tree [29].

### 2.6. High-Throughput Sequencing

To create simulated communities intended for Illumina NovaSeq 6000 sequencing, we mixed 16 DNA samples (1 µL each) from four plants (with four roots per plant) per location. This process involved 48 DNA samples collected from three distinct locations, all of which had undergone Sanger sequencing. The hypervariable ITS2 region of fungal nuclear ribosomal DNA was amplified independently for each location using the barcode-tagged fungal universal primers ITS86F/ITS4 [21,30], with two Tulasnellaceae-specific primer pairs: 5.8S-Tulngs/ITS4-Tul2 and TUG3 (5′-CATTGACTAYTTGAACGCATTG-3′)/ITS4-Tul2. The TUG3 primer was designed for this study to detect Tulasnellaceae-group TG3 fungi because of numerous mismatches with ITS86F and 5.8S-Tulngs [17]. PCR was performed in triplicate using 2× KAPA HiFi HotStart ReadyMix (Kapa Biosystems, Wilmington, DE, USA), following the manufacturer’s instructions. Purified PCR products underwent quantification using a Qubit dsDNA HS Assay Kit, and library preparation and sequencing were carried out according to the method presented by Chamara et al. [15].

Data quality was initially assessed using FastQC [31]. Subsequently, the reads were cleaned and demultiplexed into the corresponding samples using the barcode labels and the process_shortreads program of STACKS v. 2.61 [32]. The demultiplexed sequences underwent further processing using Qiime2 (v. 2022.11.1) software [33]. In Qiime2, the q2-cutadapt plugin [34] was employed to trim barcodes and primers from the raw sequencing data. Subsequently, the q2-dada2 plugin [35] was used to remove noise, singletons, and chimeras. Quality trimming was conducted to retain only high-quality sequence data for downstream analysis by removing 6 bp from both ends of sequences with a Phred quality score < 30. OTU clustering was carried out at a 97% similarity threshold using the VSEARCH algorithm [36]. To create a comprehensive and customized reference database, fungal reference sequences were downloaded from the UNITE database (v. 9.0) [37], and OTU sequences obtained by Sanger sequencing were added. Clustering these sequences at a 97% similarity threshold resulted in the identification of 91 OTUs. The identified fungi were classified as OMF, as described by Dearnaley et al. [4], Meng et al. [38], and Ogura-Tsujita et al. [39]. Contaminants, non-fungal OTUs, and those with fewer than five sequences were eliminated. The total reads for each OTU obtained using each primer combination for three locations were calculated.

### 2.7. In Vitro Symbiotic Germination

Three Tulasnellaceae isolates, frequently present in the cultivar and observed across at least two sites, were used for in vitro symbiotic germination (Table 2). Because isolate TU27 could not be obtained from *D*. Stardust ‘Firebird’, we substituted it with the isolate from *D. officinale*. Seeds were collected from three *Dendrobium* species: *D. nobile*, *D. officinale*, and *D. moniliforme* (one to three capsules) and used for symbiotic culture. These species were used because *D. nobile* and *D. moniliforme* are used for hybridization in *D*. Stardust ‘Firebird’. Before use, seed viability was confirmed to be > 80% using the 2,3,5-triphenyl tetrazolium chloride (TTC) test. Seeds were sterilized with 1% sodium hypochlorite solution for 1 min and sown on a water agar medium as described by Zhang et al. [40]. Each treatment contained six replicates and a total of 120–240 seeds. A 6 mm plug of fungal culture was inoculated, and the plates were cultured under a 12 h/12 h light/dark photoperiod at 25 ± 1 °C. Plates without fungal inoculum were used as the control. After 7 weeks of culturing, seeds were counted under a stereomicroscope, and seed germination and protocorm development were scored on a scale of 0–5 as follows: stage 0, no germination; stage 1, an enlarged embryo with ruptured seed coat; stage 2, a globular embryo (protocorm) with rhizoids; stage 3, a protocorm with an apical meristem; stage 4, emergence of the first leaf; stage 5, a seedling with a second leaf and further development (Supplementary Figure S1). Seed germination (%) per stage was calculated as follows: percentage seed germination = (number of seeds per germination stage/total number of viable seeds) × 100. Data were subjected to ANOVA followed by Tukey’s test using R statistical software (v. 4.2.1) [19].

### 2.8. Symbiotic Culture of Seedlings

The same three Tulasnellaceae isolates (Table 2) were cultured with seedlings obtained by asymbiotic culturing and used to assess seedling growth with *Dendrobium moniliforme*. Seedlings with three or four leaves were transplanted into plastic pots containing sterilized Sphagnum moss at one seedling per pot. The fungal isolates were crushed, dissolved in SDW, and inoculated into the pots (20 mL). Pots with no fungal inoculum were prepared as the control. Twenty replicates were used for each treatment. The cultures were maintained in plastic containers at 25 ± 1 °C under a 12 h/12 h light/dark photoperiod. After 4 months, fresh weight, dry weight, tiller number, plant height, root number, leaf number, root length, and leaf length were evaluated. The data were subjected to one-way ANOVA followed by Tukey’s test (*p* < 0.05) for pairwise comparisons using R statistical software (v. 4.2.1) [19].

## 3. Results

### 3.1. Assessment of Mycorrhization

**Figure 2:**
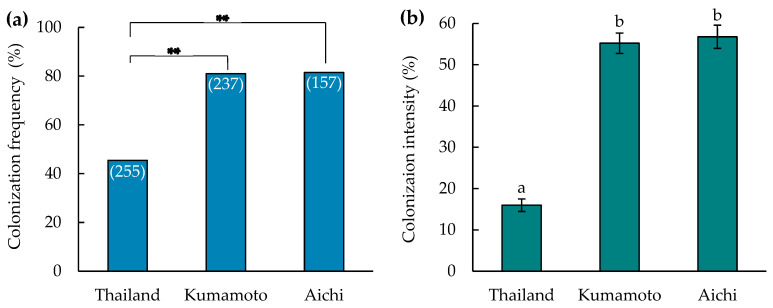
Mycorrhization of *Dendrobium* Stardust ‘Firebird’ from three locations. (**a**) Colonization frequency of mycorrhizal fungi (number of colonized root sections/total number of root sections examined) × 100 (%), with asterisks denoting significant differences (*p* ≤ 0.05, χ2 test). The total number of hand-cut sections examined per location is in parentheses. (**b**) Colonization intensity of mycorrhizal fungi (on a six-point scale of 0, 12.5%, 25%, 50%, 75%, and 100% of the cortical area). Mean values with differing letters are significantly different at *p* < 0.05 (Tukey’s test).

We examined 255, 237, and 157 root sections, representing 13 individuals from Thailand, Kumamoto, and Aichi (Table 1), respectively. Mycorrhizal colonization was consistent across all individuals. Colonization frequency was significantly lower in Thailand juveniles (45.5%) than in mature individuals from Kumamoto (81.1%) and Aichi (81.5%) (*p* < 0.05, Figure 2a). Furthermore, the colonization intensity differed significantly among individuals (*p* < 0.05, and among the three locations. Colonization intensity was significantly lower in Thailand juveniles (16%) compared to that in mature individuals from Kumamoto (55.2%) and Aichi (56.8%) (*p* < 0.05, Figure 2b). However, there was no difference in colonization frequency or intensity between mature plants from Kumamoto and Aichi. Additionally, mycorrhizal colonization was observed from the basal to the apical position and occurred mainly in the base and middle regions.

### 3.2. Molecular Identification of Mycorrhizal Fungi by Sanger Sequencing

**Figure 3:**
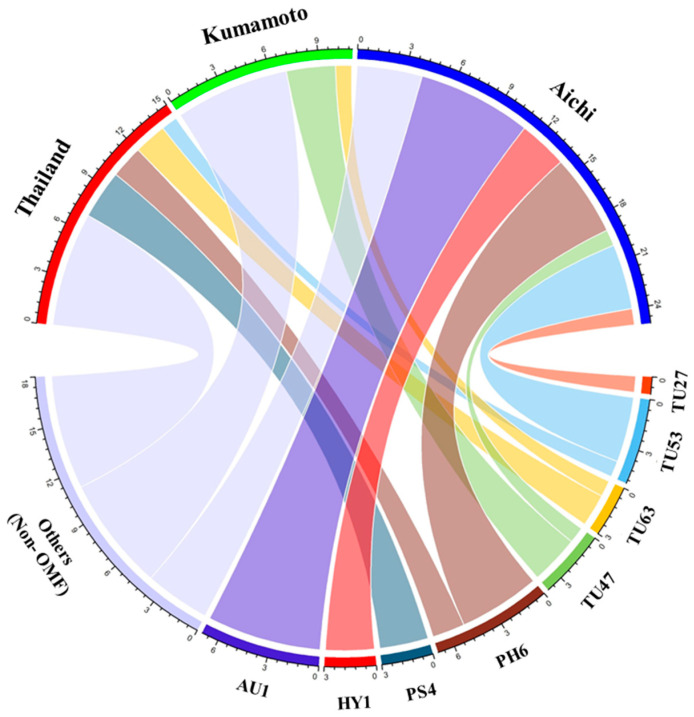
Chord diagram of the relationship between sampling locations and OTUs detected by Sanger sequencing. Strip width represents the number of OTU taxa identified. Identical sequences from a single sample using different primers were excluded. TU, Tulasnellaceae; PH, *Phlebia* sp.; PS, *Psathyrella* sp.; HY, Hymenochaetales sp.; AU, Auriculariales sp.; others, non-orchid mycorrhizal Ascomycota and Basidiomycota.

In total, 41 root samples and 3 fungal isolates collected from 13 individuals at three locations were analyzed (Supplementary Table S1). The three fungal isolates, TU27, TU47, and TU63, belonged to the Tulasnellaceae family, and each represented one OTU. In all, 51 fungal sequences were identified, 71% of which constituted OMF. The OMF were assigned to eight OTUs—Tulasnellaceae (four OTUs, 39%), *Phlebia* (one OTU, 21%), Auriculariales (one OTU, 21%), *Psathyrella* (one OTU, 9%), and Hymenochaetales (one OTU, 9%) (Figure 3). The highest number of OTUs (four) was detected in the Tulasnellaceae group; other OMF taxa exhibited one OTU each. Aichi exhibited the highest number of OTUs (n = 6). The prevalence of dominant mycorrhizal fungi varied across the three locations. *Psathyrella* (42%) was the most frequently detected OMF taxon in Thailand, and Tulasnellaceae (100%) and Auriculariales (33%) were the most frequently detected OMF taxa in Kumamoto and Aichi, respectively. PH6, TU63, and TU53 were shared by Thailand and Japan, and TU47 was detected in both Kumamoto and Aichi.

### 3.3. Phylogenetic Analysis

**Figure 4:**
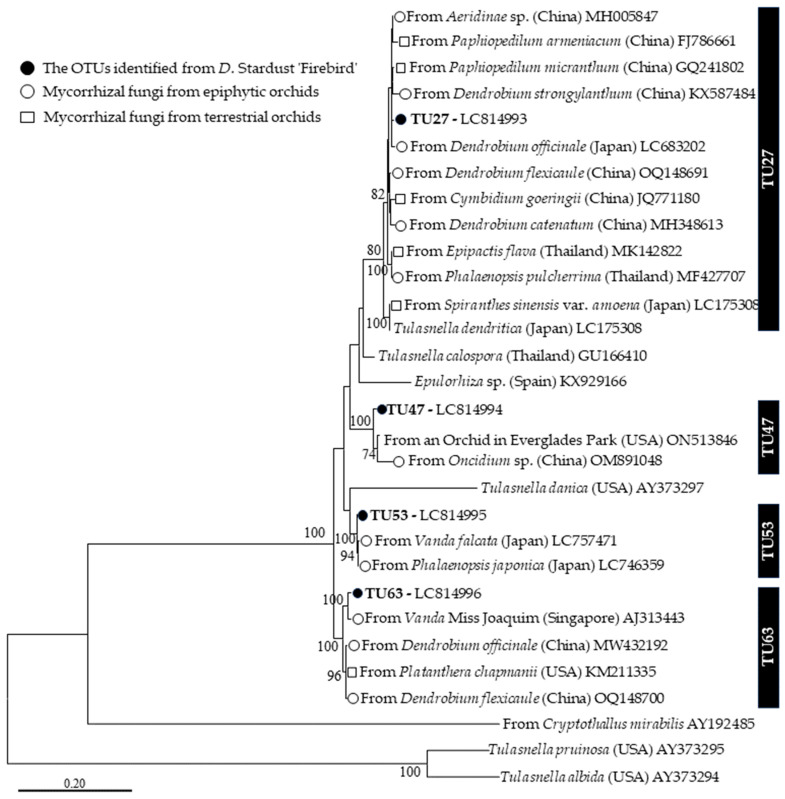
Maximum-likelihood tree of Tulasnellaceae based on the ITS1, 5.8S, and ITS2 regions, including the four OTUs identified in this study. The tree is drawn to scale; branch lengths are in the number of substitutions per site. Only bootstrap values > 70% are shown. ITS sequences of *Tulasnella pruinosa* and *Tulasnella albida* were used as the outgroup. The final dataset was 468 bp. Vertical black bars represent sequences sharing > 97% sequence similarity to each OTU.

We conducted a phylogenetic analysis of putative mycorrhizal fungi of the Tulasnellaceae family using the four OTUs identified in this study (Figure 4). The dominant mycorrhizal fungus, TU63, formed a monophyletic clade with four orchid mycobionts: a fungus from a cultivated *Vanda* Miss Joaquim (Singapore, AJ313443), and fungi from three wild orchids—*Dendrobium officinale* (China, MW432192), *Dendrobium flexicaule* (China, OQ148700), and *Platanthera chapmanii* (USA, KM211335)—which had > 97% ITS sequence similarities. Similarly, the TU53 sequence was closely related (> 97% sequence similarities) to mycorrhizal fungal sequences from the epiphytic orchids *Phalaenopsis japonica* (Japan, LC746359) and *Vanda falcata* (Japan, LC757471).

A prevalent mycorrhizal fungus, TU47, formed a monophyletic clade with orchid mycobionts found in fungi from orchids in Everglades National Park (United States; ON513846) and *Oncidium* sp. (China, OM891048), displaying > 97% sequence similarity. Additionally, the sequence of TU27 formed a monophyletic clade with *Tulasnella dendritica*, supported by a bootstrap value (BS) of 80%. This clade predominantly comprised fungal sequences from *D. officinale* (Japan), *D. strongylanthum* (China), *D. flexicaule* (China), and *D. catenatum* (China), as well as seven other orchid mycobionts from Thailand, China, and Japan (all sequence similarities > 97%).

Based on a BLAST search for non-Tulasnellaceae fungal sequences, Auriculariales (AU1) showed a 96.4% sequence similarity to a fungus found in *Pleione* species (China, MG707451). *Psathyrella* (PS4) had a 97.7% sequence similarity to a fungus identified in *Oeceoclades maculata* (Puerto Rico, KU879331). Hymeonochetales (HY1) displayed a 96.7% sequence similarity to fungi associated with *Bulbophyllum variegatum* (Reunion, JF691058). *Phlebia* (PH6) exhibited a 91.2% sequence similarity to a fungus from *Vanda falcata* (Japan, LC757472).

### 3.4. High-Throughput Sequencing

**Figure 5:**
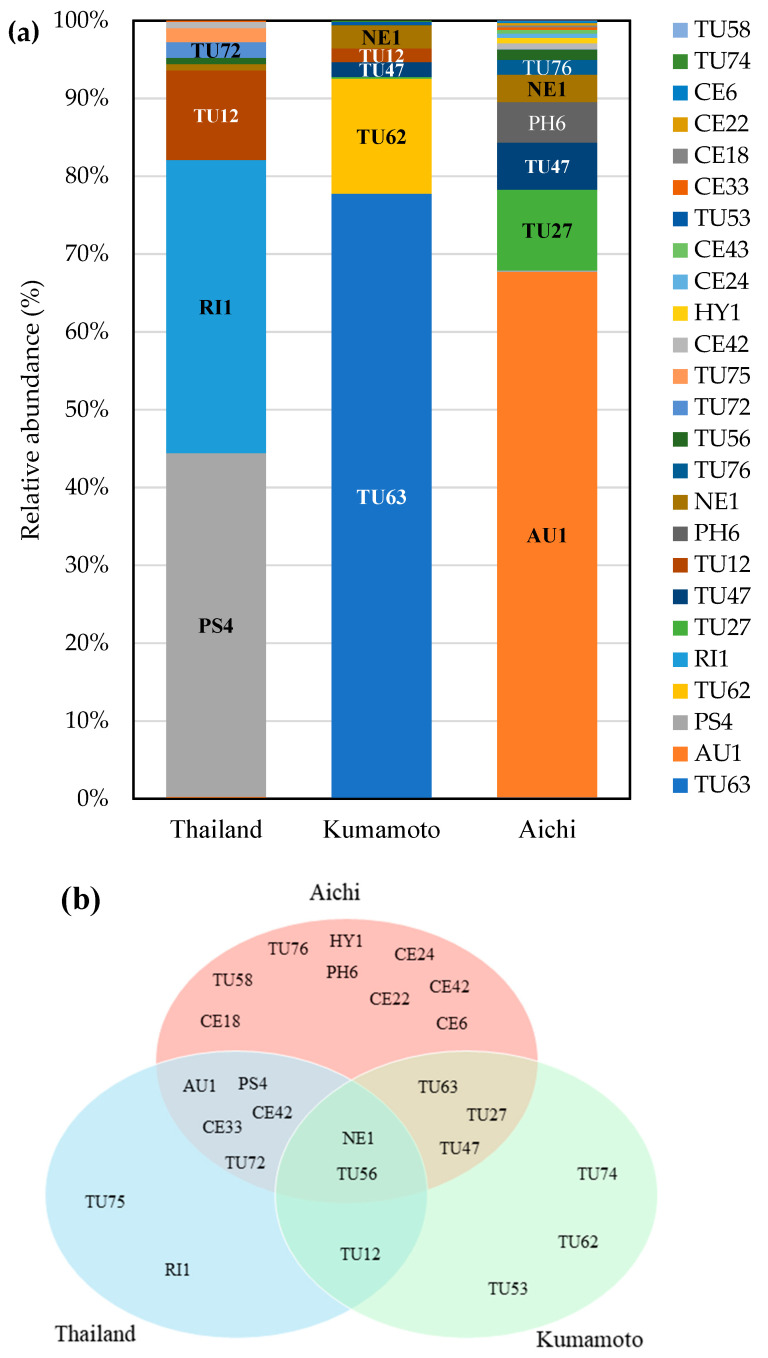
OMF detected by high-throughput sequencing. (**a**) Relative abundances of OMF OTUs from *D*. Stardust ‘Firebird’ using fungal universal (ITS86F/ITS4) and Tulasnellaceae-specific (5.8S-Tulngs/ITS4-Tul2 and TUG3/ITS4-Tul2) primers. Sequences were assigned to OTUs at 97% sequence similarity. In total, 25,999 reads were obtained for OMF. (**b**) Venn diagram is the OMF OTUs shared by the three locations. TU, Tulasnellaceae; CE, Ceratobasidiaceae; AU, Auriculariales; PS, *Psathyrella*; RI, *Rigidoporus*; NE, *Neonothopanus*; PH, *Phlebia*; HY, Hymenochaetales.

Three primer combinations were used to identify the potential OMF of *D*. Stardust ‘Firebird’, yielding a total of 38,838 raw sequence reads. After removing ambiguous and low-quality reads, 35,649 high-quality reads were retained. After eliminating contaminants, the final dataset comprised 61 fungal OTUs, totaling 33,507 sequences. Among the primers used, ITS86F/ITS4 detected 50 OTUs, and 5.8S-Tulngs/ITS4-Tul2 and TUG3/ITS4-Tul2 detected 10 and 7 OTUs, respectively. The total read count in the final OTU table was highest in Aichi (19, 696), followed by Kumamoto (12, 130) and Thailand (10, 962) (Supplementary Figure S1). Among the OTU sequences, 25 OTUs (25,999) were classified into putative OMF taxa, comprising 12 Tulasnellaceae, 1 Auriculariales, 1 *Psathyrella*, 1 *Rigidoporus*, 1 *Neonothopanus*, 1 *Phlebia*, 1 Hymenochaetales, and 7 Ceratobasidiaceae (Figure 5a).

All the OMF OTUs detected by Sanger sequencing were also detected by HTS (Figure 3 and Figure 5). HTS detected additional OMF OTUs belonging to *Rigidoporus*, *Neonothopanus*, and Ceratobasidiaceae. The main fungal partners differed among the three locations. The dominant OMF fungi identified by Sanger sequencing in each location were also dominant by HTS—Thailand, *Psathyrella* sp. (44%); Kumamoto, Tulasnellaceae (97%); and Aichi, Auriculariales (68%) (Figure 3 and Figure 5). Among the 25 OMF OTUs identified, Aichi had the highest number (19 OTUs), followed by Thailand (10 OTUs) and Kumamoto (9 OTUs). Notably, 2 OTUs (NE1 and TU56) were consistently detected at all three locations; 9 OTUs were shared by at least two sites, and 14 OTUs were exclusive to one location (Figure 5b). Moreover, eight OTUs were shared by Thailand and Japan, and five OTUs were shared between Kumamoto and Aichi.

Based on the BLAST search results, *Rigidoporus* sp. (RI1) showed 100% sequence similarity to a fungus isolated from the epiphytic orchid *Dendrobium moniliforme* (China, MN173016); 99.7% sequence similarity to a fungus isolated from a terrestrial orchid *Nervilia aragoana* (China, MH005862); and 98.7% similarity to a fungal symbiont isolated from a terrestrial orchid, *Eulophia pulchra* (Reunion Island, JF691147). Additionally, *Neonothopanus* sp. (NE1) exhibited 99.7% sequence similarity to the mycorrhizal fungi of a mycoheterotrophic orchid, *Erythrorchis altissima* (Japan, LC327057).

### 3.5. Effects of Fungal Strains on Seed Germination

**Figure 6:**
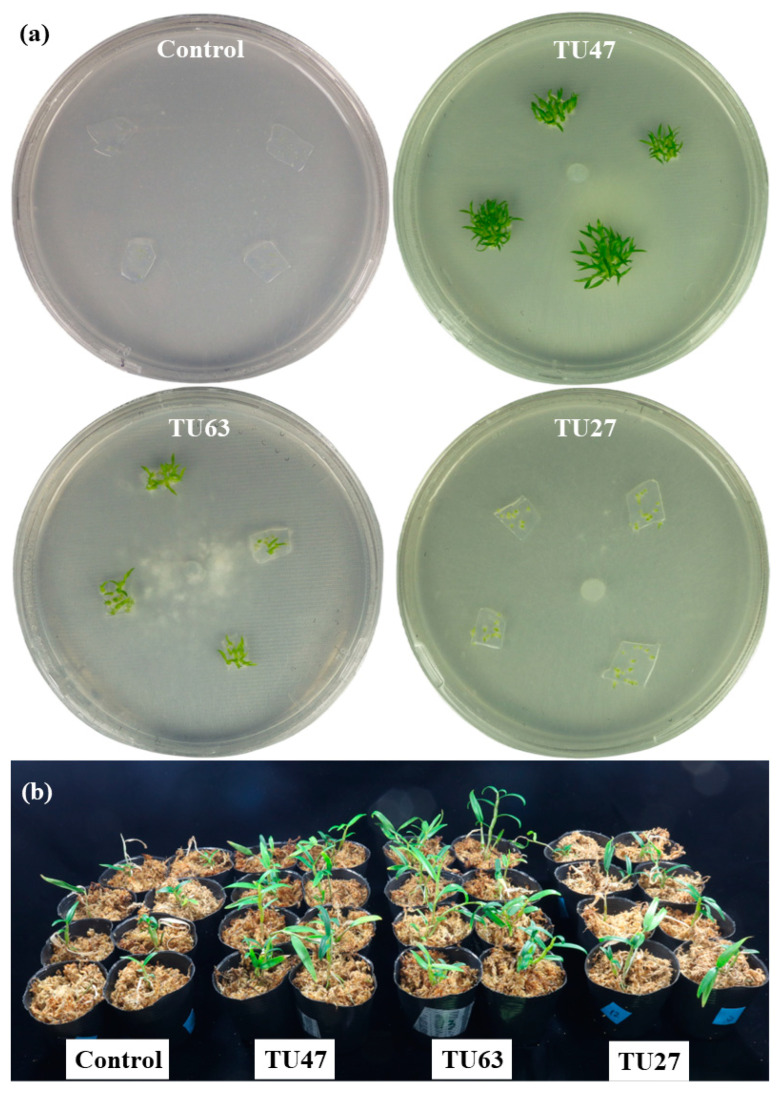
Effect of Tulasnellaceae fungi on seed germination and seedling growth in symbiotic culture. (**a**) Effects of fungal isolates on seed germination of *D. nobile* after 7 weeks. (**b**) Effects of fungal isolates on seedling growth of *D. moniliforme* after 4 months.

**Figure 7:**
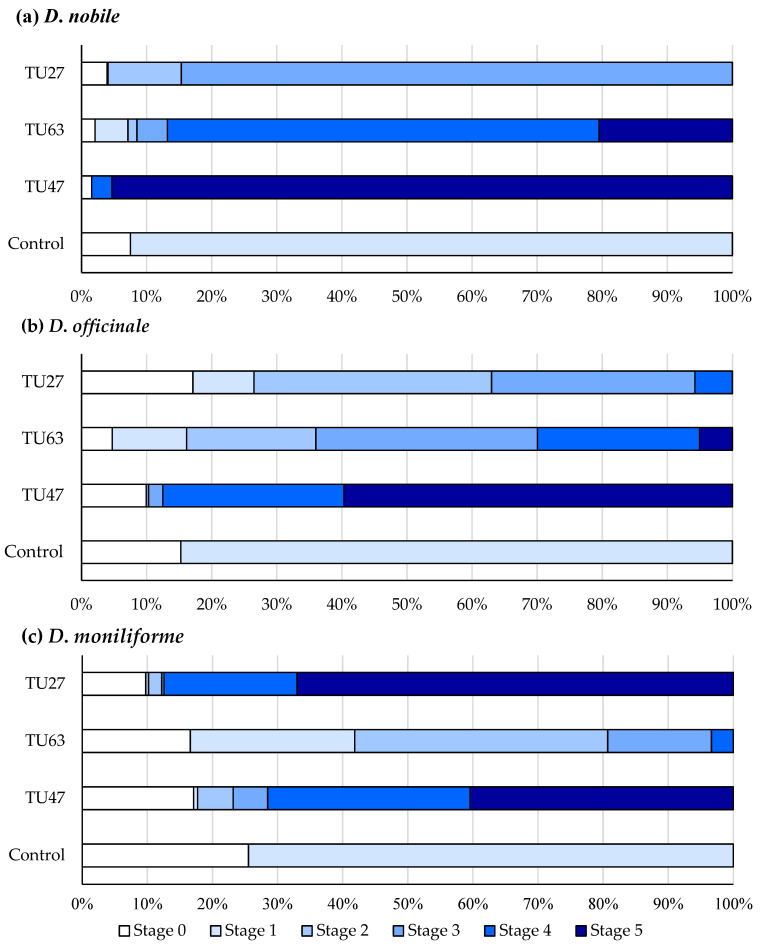
Rates (%) of seed germination and plant growth stages in *D. nobile* (**a**), *D. officinale* (**b**), and *D. moniliforme* (**c**) after 7 weeks of culture. Seed development was categorized into five stages. The control group comprised non-inoculated seeds. Data present means from six replicates.

Seeds of *D. nobile*, *D. officinale*, and *D. moniliforme* were symbiotically cultured with three Tulasnellaceae isolates for 7 weeks (Table 2). All three isolates significantly promoted seed germination and protocorm development to varying degrees (Figure 6a and Figure 7; Supplementary Table S2) compared to the control. TU47 enhanced seed germination and protocorm development up to stage 5, the highest stage achieved by the three *Dendrobium* species. Moreover, it facilitated the fastest transition to stage 5 in *D. nobile* and *D. officinale*.

TU63 significantly improved seed germination and protocorm development up to stage 5 in *D. nobile* and *D. officinale*. However, in *D. moniliforme*, TU63 advanced development only up to stage 4. TU27 exerted the most pronounced effect in *D. moniliforme*, which reached stage 5 earlier than other isolates. In *D. nobile* and *D. officinale*, TU27 led to development up to stages 2 and 4, respectively. By contrast, seeds in the non-inoculated control group became swollen with rupture of the seed coat (stage 1), but no further embryo development.

### 3.6. Effects of Fungal Strains on Seedling Growth

**Figure 8:**
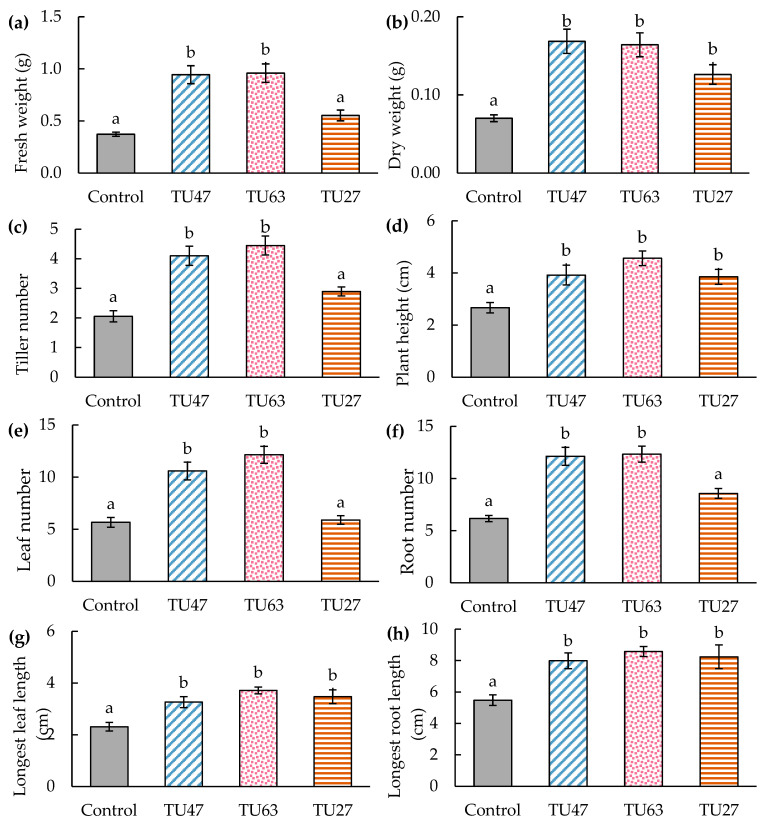
Effect of fungal inoculation on the growth of asymbiotic *D. moniliforme* seedlings at 4 months after symbiotic culture. Fresh weight (**a**), dry weight (**b**), tiller number (**c**), plant height (**d**), leaf number (**e**), root number (**f**), longest leaf length (**g**), and longest root length (**h**). Bars (means of 20 replicates ± standard error) topped by the same letter do not differ significantly at *p* < 0.05 by Tukey’s test based on one-way ANOVA.

All the seedlings survived inoculation of TU47 and TU63, whereas the control and TU27 showed 90% and 95% survival rates, respectively. All the Tulasnellaceae isolates promoted seedling growth of *D. moniliforme* (Figure 6b and Figure 8). Dry weight, plant height, leaf, and root length showed significant increases after treatment with TU63, TU47, and TU27 compared to the control. Fresh weight, tiller number, leaf, and root number were significantly higher in TU47 and TU63 compared to TU27 and the control. Dry weight was evaluated separately for shoots and roots (Supplementary Figure S3). Shoot and root dry weights differed significantly among the treatments, and TU47 showed significantly higher root dry weights than the other treatments. Microscopic examination indicated colonization of root cortical cells by all the fungal strains. Seedlings inoculated with TU47 and TU63 showed robust colonization, whereas those treated with TU27 were rarely colonized by mycorrhizal fungi.

## 4. Discussion

### 4.1. Assessment of Mycorrhization

Mycorrhizal colonization was consistently observed across all individuals from the three locations, indicating the establishment of a strong mycorrhizal association in *D*. Stardust ‘Firebird’. This is the first study to show extensive mycorrhizal colonization in an orchid cultivar in a horticultural setting. Mature plants in Aichi and Kumamoto exhibited higher colonization frequencies and intensities compared to Thailand juveniles, but there was no difference between Kumamoto and Aichi (Figure 2). The influence of plant age on colonization may be more pronounced than that of location. In New Zealand nurseries, orchid hybrids cultivated for 5–20 years showed higher mycorrhizal colonization rates than those cultivated for 2–15 months [9]. Mature plants likely have a longer period to establish a mycorrhizal association than young seedlings, and their larger and more developed root systems enable colonization by mycorrhizal fungi. Differences in climate and other environmental factors may also explain the variable colonization abundance. Mycorrhizal colonization was observed primarily at the basal to the apical positions of roots, most frequently in the base and middle regions. This finding supports a report that new roots or the growing tips of the oldest roots have little mycorrhizal colonization [41]. The apical positions, being younger and actively growing, allow more time for establishment.

### 4.2. Mycorrhizal Fungal Community of D. Stardust ‘Firebird’

Sanger sequencing and HTS indicated that *D*. Stardust ‘Firebird’ is associated with a phylogenetically diverse OMF community, as are wild orchids (Figure 3 and Figure 5). The predominant fungal OTUs were consistent across both sequencing methods, indicating no significant primer bias. The two molecular techniques identified diverse Tulasnellaceae fungi—4 OTUs were detected by Sanger sequencing and 12 OTUs by HTS. *Dendrobium* spp. are primarily associated with Tulasnellaceae fungi [25,40,42]. Our findings indicate that hybrid *Dendrobium* is predominantly associated with Tulasnellaceae fungi. The phylogenetic analysis showed that Tulasnellaceae fungi associated with *Dendrobium* hybrids under cultivated conditions are distributed globally and are common among wild orchids (Figure 4). Specifically, TU27, TU47, and TU63 are distributed worldwide, having been detected in China, Japan, Thailand, Singapore, and the United States. These three Tulasnellaceae OTUs are associated with wild orchid species, and TU27 and TU63 are prevalent in epiphytic and terrestrial orchids. Additionally, TU63 was identified in a hybrid orchid, *Vanda* Miss Joaquim, implying that this OTU is common under cultivated conditions. OMF in cultivated environments may originate from the surrounding area or other nurseries where plantlets are cultured, or from the culture medium.

Non-Tulasnellaceae fungi predominated among those associated with *D*. Stardust ‘Firebird’. *Psathyrella* (PS4; LC814997), *Phlebia* (PH6; LC815000), Auriculariales (AU1; LC814999), and *Rigidoporus* (RI1) were prevalent by Sanger sequencing or HTS (Figure 3 and Figure 5). These fungi showed high sequence similarities with those from orchid species, implying a symbiotic relationship with *D*. Stardust ‘Firebird’. *Psathyrella* fungi, which are major non-*Rhizoctonia* OMF, have been found in terrestrial orchids, such as *Oeceoclades maculata* [43], *Eulophila zollingeri* [44], and *Cremastra appendiculata* [45]. Similarly, *Phlebia* and *Neonothopanus* fungi are associated with the mycoheterotrophic orchid, *Erythrorchis altissima* [39]. *Psathyrella* [46], *Phlebia* [47], *Neonothopanus* [48], and *Rigidoporus* [49] are linked to wood decay. The presence of wood-decay fungi in *D*. Stardust ‘Firebird’ is likely a result of the composition of the medium, particularly the inclusion of coconut husk, which differs from the nutrient conditions of wild orchids. *Vanilla planifolia* cultivated on bagasse substrate was predominantly associated with the wood-decay fungus *Resinicium saccharicola*, but with Tulasnellaceae fungi under other cultivation conditions [50]. Associations with wood-decay fungi may be unique to cultivated orchids because of the different growth substrates. Although these wood-decay fungi could be mycorrhizal, further experiments, such as symbiotic culture, are needed to reach a definitive conclusion. As the use of wood-decay fungi, such as *Pleurotus ostreatus* and *Lentinula edodes*, significantly improves the survival and growth of *Cattleya* hybrid seedlings [51,52], wood-decay fungi may have potential as growth promoters. In addition to wood-decay fungi, HTS identified seven Ceratobasidiaceae OTUs that were not detected by Sanger sequencing. Ceratobasidiaceae, an important OMF group in orchids, may also be associated with *D*. Stardust ‘Firebird’.

### 4.3. Variation in Mycorrhizal Community Composition

The two molecular techniques revealed variations in the OMF communities among the three nurseries. The fungal OTUs PS4, TU47, and AU1 were predominantly detected in Thailand, Kumamoto, and Aichi, respectively, by Sanger sequencing (Figure 3), whereas the relative abundances of PS4, TU63, and AU1 were the highest in Thailand, Kumamoto, and Aichi, respectively, by HTS (Figure 5a). Although the Aichi nursery imported potted seedlings from the Thailand nursery, there was a marked difference in the dominant fungal OTUs between the two locations. These findings indicate that the mycorrhizal community is considerably influenced by the growth environment. Mature plants cultured in Japan (Kumamoto and Aichi) had markedly different fungal communities between sites by both Sanger sequencing and HTS, and the diversities were higher in Aichi. This implies that the growth environment, rather than the growth stage, determines the composition of the fungal community. Mycorrhizal communities of wild orchids are influenced by their geographic location and local environmental conditions [53,54]. Orchid cultivation practices, including substrate and method, significantly affect the fungal community [50]. Further research should evaluate the factors that influence the OMF community during cultivation, including the effects of irrigation techniques, temperature variations, growth medium, and management practices.

Although the dominant fungal OTUs differed among the three sites, several were detected at both sites by both Sanger sequencing and HTS. Two OTUs were detected by Sanger sequencing (Figure 3), and seven OTUs by HTS (Figure 5b) in Thailand and Aichi, indicating that the associated fungi were retained after the seedlings were imported from Thailand. A terrestrial orchid, *Spiranthes spiralis*, continued to associate with the same OMF after being transplanted into a pot with new soil despite the replacement of the primary OMF [55]. These fungi were retained after transplanting, implying that they benefit plant growth.

### 4.4. Effects of Fungi on Seed Germination and Seedling Growth

Our results show that the Tulasnellaceae fungi associated with *D*. Stardust ‘Firebird’ promote seed germination and growth of *Dendrobium* spp. Additionally, inoculation of Tulasnellaceae fungi enhanced the survival rate of asymbiotic seedlings, implying that they promote the growth of cultivated hybrids. The three Tulasnellaceae isolates promoted seed germination in three *Dendrobium* species, and TU47 promoted seed germination in all three *Dendrobium* species up to stage 5. These findings indicate that the dominant Tulasnellaceae fungi associated with *D*. Stardust ‘Firebird’ have a broad range of host compatibility. The host range of OMF can vary markedly. For instance, Bonnardeaux et al. [56] examined the compatibility of 11 OMF isolates with six orchid species; the compatible host range varied from one to three hosts. Zettler and Dvorak [57] reported that a *Tulasnella calospora* strain facilitated the germination of the seeds of 39 orchid species of 21 genera. Because horticultural hybrids are typically created by crossing orchid species, OMF associated with orchid cultivars may have a broad host range.

The three Tulasnellaceae fungi significantly promoted seed germination and seedling growth in *D. moniliforme*, suggesting that they exert their effects at multiple stages of the plant lifecycle. Orchid seed germination relies on mycorrhizal fungi for carbon resources. However, once leaves have developed, plants obtain carbon by photosynthesis and rely on fungi to supply other essential nutrients (e.g., nitrogen and minerals). Because the physiological requirements of plants shift after leafing, the fungi necessary for plant development may also change [58]. In fact, promotive fungal strains differed from seed germination to seedling growth in *D. okinawense* [16] and *Vanda falcata* [12]. Nonetheless, some fungal OTUs are effective at both the seed germination and seedling growth stages in *D. moniliforme*, *D. officinale* [16,59], and *D. exile* [60]. Notably, TU47 and TU63 were isolated from mature plants in Kumamoto and juveniles in Thailand, respectively. Therefore, these fungi may be effective throughout the lifecycle of *D*. Stardust ‘Firebird’.

## 5. Conclusions

To our knowledge, this is the first comprehensive assessment of the mycorrhizal associations of a horticultural orchid hybrid under cultivation conditions. We investigated a single cultivar from three nurseries; diverse mycorrhizal fungi colonized the *Dendrobium* cultivar at a high colonization rate. Similar to wild orchids, our findings indicate that hybrid orchids are likely to be frequently associated with multiple OMF. Moreover, the mycorrhizal fungi associated with the orchid cultivar significantly promoted seed germination and seedling growth in multiple *Dendrobium* species. Such fungal species have potential as growth promoters for commercial production. Indeed, those with a broad range of host plants, such as TU47, have considerable potential for such applications because most horticultural cultivars are produced by hybridizing multiple orchid species. In addition, it is advantageous to use fungal strains that support plant growth irrespective of the growth stage. Furthermore, because orchid seeds germinate symbiotically more often than asymbiotically [61,62], the use of mycorrhizal fungi for seed germination could shorten the breeding period of orchid hybrids. In this study, we identified abundant wood-decay fungi in the roots of *D*. Stardust ‘Firebird’. Wood-decay fungi associated with *D*. Stardust ‘Firebird’ may have the potential to promote the growth of cultivated orchids. Finally, future studies should explore more orchid cultivars and genera to determine whether other hybrid orchids in horticulture have orchid mycorrhizal associations.

## Figures and Tables

**Table 1:** Details of sampling locations, including the numbers of individuals, roots, and sections analyzed, together with growth stage, and growth medium.

Location	No. of Plants	No. of Roots	No. of Sections Observed	Growth Stage	Growth Medium
Chiang Mai Prov., Thailand	5	64	255	Juvenile	Coconut husk
Kumamoto Pref. Japan	4	46	237	Mature	Sphagnum moss
Aichi Pref. Japan	4	28	157	Mature	Coconut husk and Sphagnum moss

Abbreviations: Prov., province; Pref., prefecture.

**Table 2:** Tulasnellaceae isolates from *Dendrobium* Stardust ‘Firebird’ and *D. officinale* used for symbiotic culture.

Fungal OTU	Host Orchid	DDBJ Accession No.	NBRC Accession No.
TU27	*D. officinale*	LC683202	NBRC 115262
TU47	*D*. Stardust ‘Firebird’	LC814994	NBRC 116648
TU63	*D*. Stardust ‘Firebird’	LC814996	NBRC 116574

Abbreviations: OTU, operational taxonomic unit; DDBJ, DNA Data Bank of Japan; NBRC, National Institute of Technology and Evaluation (NITE) Biological Resource Center.

## Data Availability

The sequence reads have been deposited into the DNA Data Bank of Japan (DDBJ) under the accession numbers LC814993–LC815000. All other data and materials supporting this article are available from the corresponding author, Y.O.-T., upon reasonable request.

## Supplementary Materials

The following supporting information can be downloaded at: https://www.mdpi.com/article/10.3390/microorganisms12061176/s1, Table S1: The relationship between various sampling locations and the detected OTUs obtained through Sanger sequencing; Table S2: Effects of fungal isolates on germination and protocorm development of three *Dendrobium* species (*D. nobile* (a), *D. officinale* (b), and *D. moniliforme* (c) after seven weeks of culturing; Figure S1: Stacked bar graph shows absolute read counts of OMF OTUs from *D*. Stardust ‘Firebird’ using fungal universal (ITS86F/ITS4) and Tulasnellaceae-specific (5.8S-Tulngs/ITS4-Tul2 and TUG3/ITS4-Tul2) primer pairs; Figure S2: The six developmental stages of seed germination and protocorm development in *Dendrobium nobile*; Figure S3: Effect of fungal inoculation on the growth of asymbiotic *Dendrobium moniliforme* seedlings at 4 months after symbiotic culture. Shoot dry weight (a), root dry weight (b).
