# Supplementary material for: Orchid Mycorrhizal Association of Cultivated Dendrobium Hybrid and Their Role in Seed Germination and Seedling Growth
Source: Microorganisms. 2024 Jun 10;12(6):1176. doi: 10.3390/microorganisms12061176 (PMC11205499; doi:10.3390/microorganisms12061176)
Supplement: Supplementary file 1 [file microorganisms-12-01176-s001.zip › microorganisms-3048225-supplementary.pdf]

Supplementary Table S1. The relationship between various sampling locations and the detected OTUs obtained through Sanger sequencing. Identical sequences derived from a single sample using different primer pairs were excluded. TU: Tulasnellaceae, PH: *Phlebia* sp., PS: *Psathyrella* sp., HY: Hymenochaetales sp., AU: Auriculariales sp., Others: Non-mycorrhizal Ascomycota and Basidiomycota.

Location	Plant ID	No of DNA Samples		Fungi identified								
		Roots	Isolates	TU27	TU47	TU53	TU63	PH6	PS4	HY1	AU1	Others
Thailand	1	6	1			1	1	2				4
	2	2							1			1
	3	3							2			1
	4	2					1					1
Kumamoto	1	2	1		1							1
	2	4			1							3
	3	3			1							3
	4	4					1					
Aichi	1	5	1	1		1		3				
	2	2				1		2			2	3
	3	4			1	1				2	3	
	4	4				1				1	2	1

Supplementary Table S2. Effects of fungal isolates on germination and protocorm development of three *Dendrobium* species (*D. nobile* (a), *D. officinale* (b), and *D. moniliforme* (c) after seven weeks of culturing**.** Germination percentage (mean ± SE, n=6) within columns marked by different letters are significantly different at $p < 0.05$ (Tukey's test).

Treatment	Ratio of seed germination and protocorm development (%)					
(a) *Dendrobium nobile*						
	Stage 0	Stage 1	Stage 2	Stage 3	Stage 4	Stage 5
TU27	3.90±1.18ab	0.20±0.16a	11.3±2.03b	84.7±2.08b	0.00±0.00a	0.00±0.00a
TU63	2.10±0.08a	5.10±5.06a	1.40±0.51a	4.70±3.31a	66.3±5.59b	20.5±6.24b
TU47	1.60±0.16a	0.00±0.00a	0.00±0.00a	0.00±0.00a	3.10±0.65a	95.3±0.51c
Control	7.50±0.86b	92.5±0.86b	0.00±0.00a	0.00±0.00a	0.00±0.00a	0.00±0.00a
(b) *Dendrobium officinale*						
	Stage 0	Stage 1	Stage 2	Stage 3	Stage 4	Stage 5
TU27	17.1±4.33a	9.40±2.74ab	36.5±7.21c	31.2±6.57b	5.80±5.76a	0.00±0.00a
TU63	4.70±1.41a	11.4±5.06b	19.8±3.09b	34.0±5.88b	24.9±1.83b	5.10±3.09a
TU47	9.90±4.02a	0.00±0.00a	0.40±0.35a	2.20±1.47a	27.8±6.39b	59.7±7.79b
Control	15.3±1.77a	84.7±1.77c	0.00±0.00a	0.00±0.00a	0.00±0.00a	0.00±0.00a
(c) *Dendrobium moniliforme*						
	Stage 0	Stage 1	Stage 2	Stage 3	Stage 4	Stage 5
TU27	09.7±2.55a	0.04±0.44a	02.0±1.30a	00.3±0.35ab	20.4±4.22b	67.0±3.55c
TU63	16.6±2.20bc	25.3±8.57b	38.9±6.47b	15.9±5.30c	03.4±1.75a	00.0±0.00a
TU47	17.1±2.40bc	00.6±0.62a	05.5±2.12a	05.3±1.88abc	31.1±3.03c	40.4±4.65b
Control	25.5±1.35c	74.5±1.35c	00.0±0.00a	00.0±0.00a	00.0±0.00a	00.0±0.00a

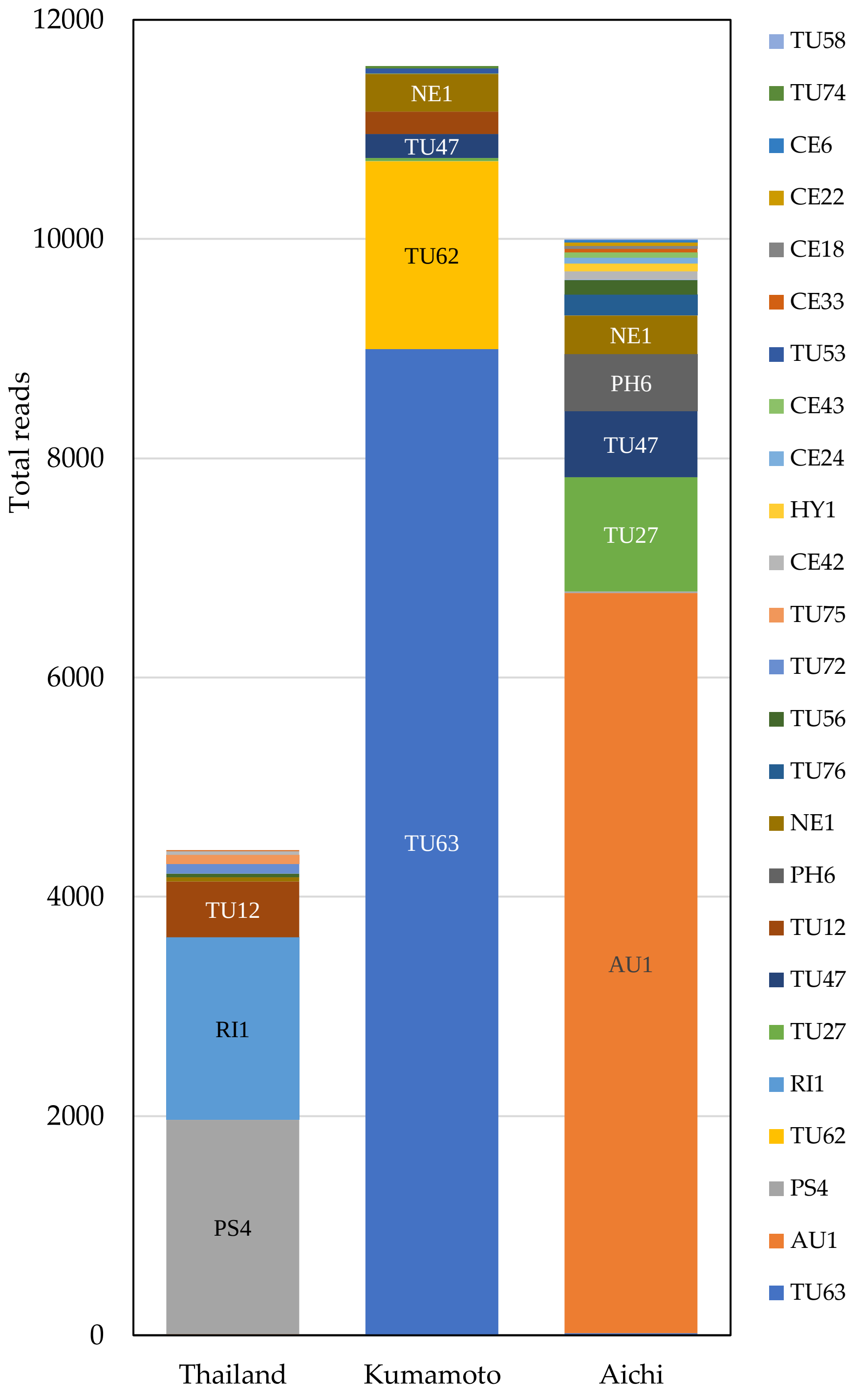

Supplementary Figure S1. Stacked bar graph shows both absolute read counts of OMF OTUs from *D.* Stardust 'Firebird' using fungal universal (ITS86F/ITS4) and Tulasnellaceae-specific (5.8S-Tulngs/ITS4-Tul2 and TUG3/ITS4-Tul2) primer pairs. OTUs with fewer than 5 total reads were excluded. TU: Tulasnellaceae, CE: Ceratobasidiaceae, AU: Auriculariales, PS: *Psathyrella*, RI: *Rigidoporus*, NE: Neonothopanus, PH: *Phlebia* and HY: Hymenochaetales. Sequences were assigned to OTUs at 97% sequence similarity. In total, 25, 999 reads were obtained for OMF fungi.

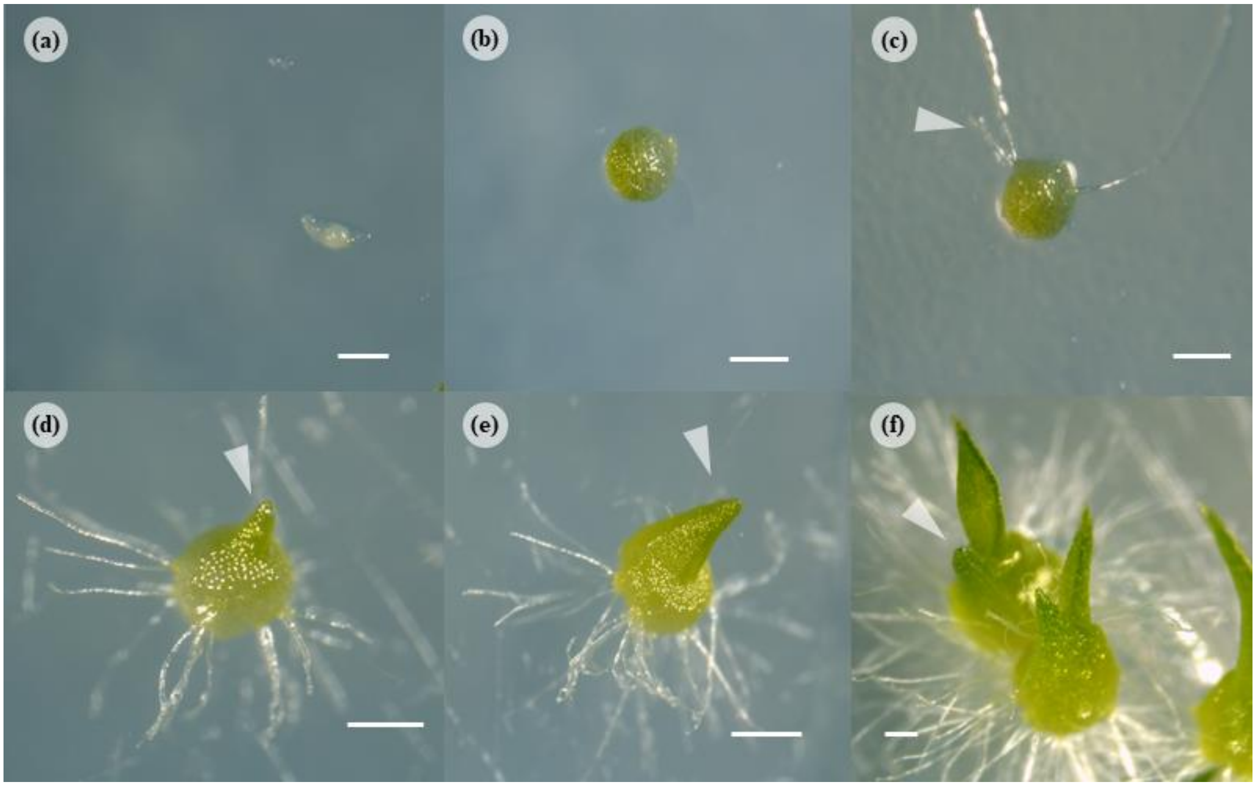

Supplementary Figure S2. The six developmental stages of seed germination and protocorm development in *Dendrobium nobile* (a) stage 0: no germination; (b) stage 1: an enlarged embryo with ruptured seed coat; (c) stage 2: a globular embryo (protocorm) with rhizoids (arrow); (d) stage 3: a protocorm with an apical meristem (arrow); (e) stage 4: emergence of the first leaf (seedling formation); and (g) stage 5: a seedling with a second leaf and further development. Bar 0.25mm.

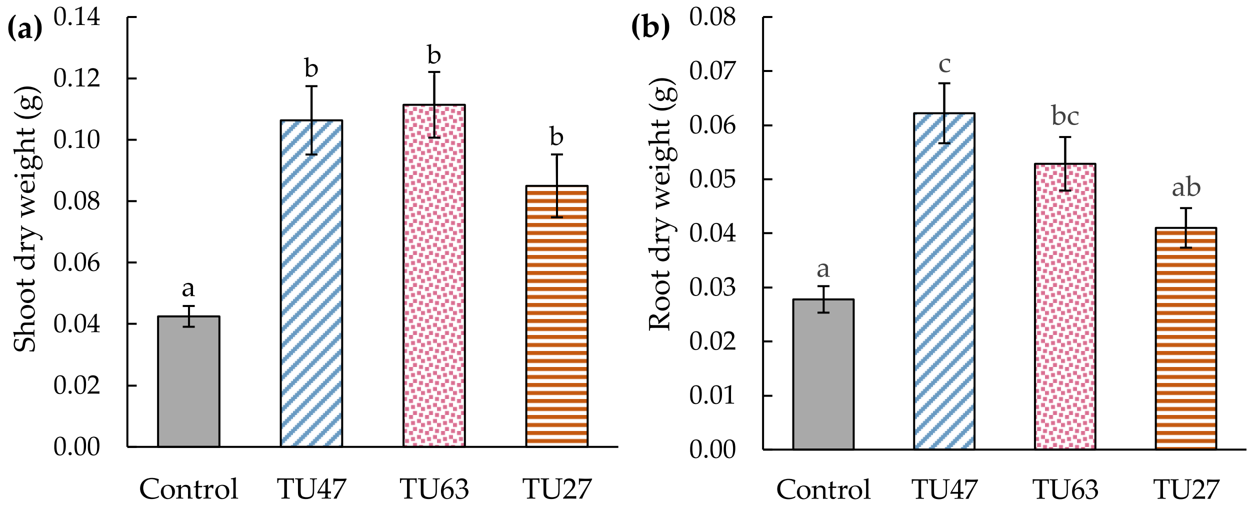

Supplementary Figure S3. Effect of fungal inoculation on the growth of asymbiotic *Dendrobium moniliforme* seedlings at 4 months after symbiotic culture. Shoot dry weight (a), root dry weight (b). Bars (means of 20 replicates ± standard error) topped by the same letter do not differ significantly at $p < 0.05$, as shown by Tukey's test based on the one-way ANOVA.
